# Current and Future Treatment Strategies for Esophageal Adenocarcinoma

**DOI:** 10.3390/cancers12102930

**Published:** 2020-10-12

**Authors:** Alexander Quaas

**Affiliations:** Institute for Pathology, University of Cologne, 62 Kerpener Street, D-50937 Cologne, Germany; alexander.quaas@uk-koeln.de

**Keywords:** esophageal adenocarcinoma, treatment, molecular targets, matrixome, immune checkpoint inhibitors

The incidence of adenocarcinoma of the esophagus (EAC) is increasing worldwide. In some regions of Europe, more adenocarcinomas are now treated than squamous cell carcinomas. In the Asian region, squamous cell carcinoma is clearly dominant, but adenocarcinoma is also diagnosed there more often [1,2].

Neoadjuvant therapy regimens, such as the CROSS scheme, have slightly improved the prognosis in potentially curatively-treatable patients over the last 10 years. Especially patients with adenocarcinoma benefit less significantly compared to those with squamous cell carcinoma. In the case of recurrence, hardly any personalized therapy options are available and unfortunately, only for a part of the patients is a curatively-intended therapy possible at all when the diagnosis is made. The choice of appropriate palliative therapeutics or other palliative measures is of high practical relevance [3].

At the molecular and therapeutic level, there are similarities and relevant differences between the adenocarcinoma of the stomach and the adenocarcinoma of the esophagus. While the adenocarcinoma of the stomach is molecularly divided into four subtypes (Chromosomal instable (CIN), Microsatellite instable (MSI), Epstein-Barr-Virus-related (EBV), Genomic stable (GS)), the adenocarcinoma of the esophagus shows, almost exclusively, the molecular characteristics of the CIN subtype, which is characterized by a TP53 mutation and a high number of copy number variations (including amplifications of ERBB2 and KRAS). In contrast to the stomach, the MSI subtype is very rarely found in the esophagus (20% versus 1%) and the EBV subtype not at all. The two latter subtypes are relevant with regard to a therapy prediction of immune checkpoint inhibitors (ICI). The molecular characteristics of the approximately 10% ICI-sensitive EACs are currently being analyzed [4].

One of the peculiarities is the fact that 70–90% of EACs occur in men. It is completely unclear why it is predominantly men who develop the carcinoma, while the main risk factor, Barrett’s mucosa, occurs only twice as frequently at most in men. What factors promote the malignant transformation of Barrett’s mucosa in men [5,6]?

How can I use newly gained molecular insights to successfully apply personalized therapeutics for defined subgroups? What is the composition of the inflammatory micromileu of EAC? Are there definable subgroups beyond PD-L1 expression that particularly benefit from immunocheckpoint blockade [7]? When is the ideal time for a (combinatorial) administration of immunocheckpoint inhibitors? What is the composition of the remaining tumor micromileu, which subtypes of carcinoma-associated fibroblasts (CAF) are present in EAC and can they be influenced therapeutically? What specific interactions exist between the composition of the tumor matrixome and the vessel width? What relevance can drugs, such as Losartan, which regulate vessel width play in EAC? 

This Special Issue on Current and Future Treatment Strategies for Esophageal Adenocarcinoma intends to bring together the current state of knowledge of EAC, to look beyond the horizon and to raise relevant questions that need to be solved in the future. Both basic scientists and clinicians will have their say.
